# Hepatosplenic mucormycosis post autologous stem cell transplant

**DOI:** 10.12669/pjms.333.12311

**Published:** 2017

**Authors:** Samia Yasmeen, Omer Waqas, Javeria Munir, Faisal Sultan, Abdul Hameed

**Affiliations:** 1 Dr. Samia Yasmeen, MBBS, FCPS(Medicine). Fellow Medical Oncology, Department of Medical Oncology, Shaukat Khanum Memorial Cancer Hospital & Research Centre, Lahore, Pakistan; 2 Dr. Omer Waqas, MBBS, FCPS(Histopathology). Fellow Cytopathology, Department of Pathology, Shaukat Khanum Memorial Cancer Hospital & Research Centre, Lahore, Pakistan; 3 Dr. Javeria Munir, MBBS, FCPS(Diagnostic Radiology). Fellow Interventional Radiology, Department of Radiology, Shaukat Khanum Memorial Cancer Hospital & Research Centre, Lahore, Pakistan; 4 Dr. Faisal Sultan, MBBS, FRCP, FCPS. Diplomate American Board of Internal Medicine, Consultant Physician and CEO, Department of Infectious Diseases, Shaukat Khanum Memorial Cancer Hospital & Research Centre, Lahore, Pakistan; 5 Dr. Abdul Hameed, MBBS, MD, FRCP. Clinical Hematologist and Head of Department, Department of Medical Oncology, Shaukat Khanum Memorial Cancer Hospital & Research Centre, Lahore, Pakistan

**Keywords:** Mucormycosis, Hepatosplenic, Hematopoietic stem cell transplant

## Abstract

Mucormycosis is a life threatening fungal infection and remains an important cause of morbidity and mortality in immunocompromised patients after hematopoietic stem cell transplant. We report here a case of hepatosplenic mucormycosis in a patient after autologous stem cell transplant. A young man with anaplastic large cell lymphoma underwent autologous hematopoietic stem cell transplant after achieving complete remission with standard chemotherapy and consolidative radiotherapy. He was found to have incidental hepatosplenic hypodensities on follow up imaging, that were proved to be mucormycosis on histopathology after getting CT-guided biopsy of splenic lesions. He was treated with intravenous amphotericin-B followed by complete radiological resolution of hepatosplenic lesions. Although these infections are often life threatening but limited disease may have better outcome if diagnosed and treated early and aggressively.

## INTRODUCTION

Hematopoietic stem cell transplant [HSCT] recipients have a high risk of acquiring invasive fungal infection [IFI] by virtue of prolonged myelosuppression.^1^ Mucormycosis is a devastating invasive fungal disease whose incidence has increased during the past decade.^2^,^3^ This mold belongs to the order Mucorales, which includes Mucor, Rhizopus, and Absidia.^4^-^6^

Mucormycosis now represents a major threat in transplant recipients, accounting for 2% and 8% of invasive fungal infections in recent cohorts of solid-organ and allogeneic stem-cell transplant recipients, respectively,^2^ with an overall 1-year survival of less than 20%.^2^ The most common form is rhino cerebral followed by pulmonary, cutaneous, gastrointestinal and disseminated. In the disseminated form, the most commonly involved organ is the lung followed by brain, kidney, heart and spleen.^3^-^5^,^7^,^8^ Isolated splenic involvement in the absence of systemic infection is extremely rare.^8^ Hepatic involvement of mucormycosis is also a rare event and is usually thought to arise from gastrointestinal disease, although dissemination uncommonly occurs from other sources.^9^,^10^

Mucormycosis most often occurs late [>3 months after transplantation], although cases occurring early have been observed. The possible portal of infection seems to be haematogenous or contiguous spread.^2^ High index of clinical suspicion and characteristic CT findings lead to early diagnosis.^8^ Histological examination of biopsied tissue is the preferred method of diagnosis. Invasion seen on histopathology is needed for confirmation.^5^

The mainstay of treatment is antifungal therapy with an Amphotericin B, surgery, and correction of the underlying medical condition if possible.^5^ The highest mortality is seen with disseminated disease and the lowest is seen in infections confined to skin and subcutaneous tissue.^8^

## CASE REPORT

Here we discuss the outcome of a young patient with hepatosplenic Mucormycosis, after autologous stem cell transplant. A 42 year old male presented with a diagnosis of stage IVBE, ALK negative anaplastic large cell lymphoma [ALCL]. He was treated with eight cycles of chemotherapy consisting of CHOP/IT and consolidative radiotherapy to initial site of bulky disease followed by complete metabolic resolution of disease on end of treatment PET scan. He was planned for high dose therapy [HDT] and autologous stem cell transplant [ASCT]. After two sessions of apharesis, 2.4 million cells / kg body weight were collected. High dose therapy [BEAM] was given followed by reinfusion of stem cells. His post-transplant period was complicated by neutropenic colitis that was managed appropriately. He was discharged on 19th post-transplant day with full engraftment. Restaging scans post HDT /ASCT was performed on day 100, which showed interval development of multiple focal hepatic and splenic hypo densities [Fig.1: A & B] for which a CT guided splenic biopsy was taken.

**Fig.1:**
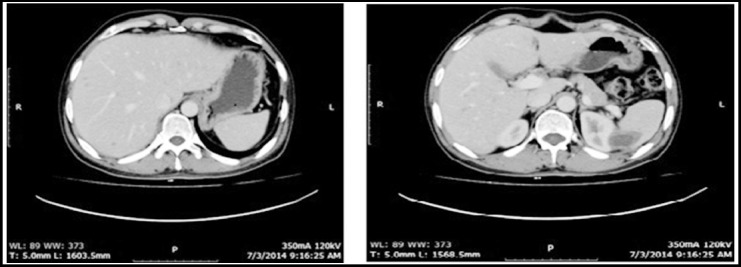
(A and B): CT neck, chest, abdomen and pelvis with contrast showed interval development of multiple focal hepatic and splenic hypodensities.

Histopathology showed necrotic debris with numerous non septate ribbon like PAS/GMS positive fungal hyphae suggestive of mucormycosis [Fig.1: C & D].

**Fig.1C:**
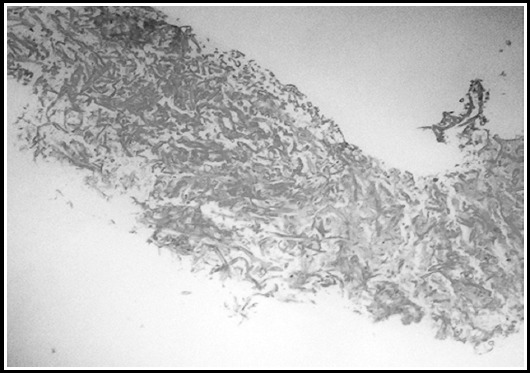
Haematoxylin and eosin [H&E] staining on splenic biopsy at light microscopy at 10x showing necrotic debris with numerous non septate ribbon like fungal hyphae at magnification 40x.

**Fig.1D:**
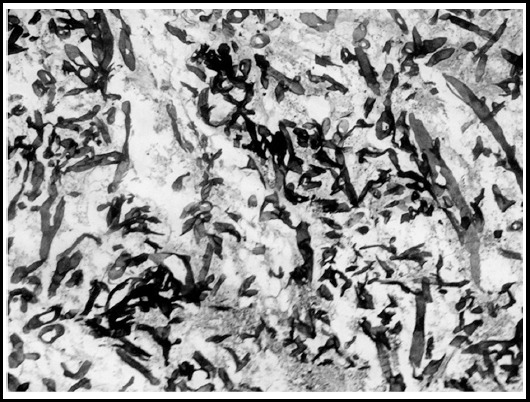
GMS staining at 40 X diffusely positive for fungal hyphae.

Fig.1D: He was started on intravenous conventional amphotericin at dosage of 1.5 mg/kg/day. But after four days of therapy he developed renal impairment with rising creatinine. He was switched to Itraconazole 200 mg twice per day which he received for a further two months. His follow-up CT scan showed interval progression in splenic and hepatic hypo-densities. He was again switched to amphotericin at the dosage of 0.7 mg/kg/day for another one month but during this period he again developed amphotericin induced nephrotoxicity. The dose of amphotericin was further reduced to 0.5 mg/kg/day with targeted total cumulative dosage of 250 mg and his renal functions were closely monitored. Follow up CT scan showed compete resolution of hepatic hypo-densities but stable appearance of splenic hypo-densities. He was discharged from hospital with follow up as outpatient with plans for a splenectomy if there was incomplete resolution of the hypodensities in the spleen. Patient remained completely asymptomatic during this whole follow-up period. His subsequent USG scan showed interval decrease in size of splenic hypo-densities that were finally resolved on USG scan [11 months from diagnosis of mucormycosis]. Currently he is doing well and remains in remission.

## DISCUSSION

Mucormycosis is a rare fungal infection, particularly seen in immunocompromised patients. This mold belongs to Mucorales family. Invasive aspergillosis is the commonest fungal infection in stem cell transplant setting followed by candidiasis and mold infections. In the liver, common fungal infection is candidiasis. Mold infections are associated with worse outcome compared other fungal infections. Post ASCT, invasive fungal infection [IFI] usually occurs in pre-engraftment period however can happen after count recovery as well. Mucormycosis is a late complication in transplant patients, often seen beyond 90 days of transplant and associated with significant morbidity and mortality. Fungal infections produce nodular infiltrates or masses in the organs. Amphotericin B and surgery, in selected cases, are major treatment options. Liposomal Amphotericin and lipid formulations are less toxic than conventional amphotericin and therefore, are preferred choices. Duration of the treatment varies according to disappearance of symptoms and signs. In this particular case, onset of mucormycosis was relatively in early period in time scale. Hepatosplenic mucormycosis is uncommon in transplant patients. Because of multiple lesions, surgery was not an option in this particular case. Fortunately, after prolonged treatment with amphotericin, all lesions disappeared. The patient remains in good health, being three years post initial presentation with the diagnosis of ALCL.

In conclusion, although mucormycosis is a serious and could be fatal in significant number of patients, but early detection and appropriate treatment may lead to excellent outcome. Although, liposomal and other lipid formulations of amphotericin are better choices, but due to cost, in many developing countries they may not be readily available. In this situation, conventional amphotericin can be started as soon as a patient is diagnosed with mucormycosis.

### Authors’ Contribution

***SY & AH:*** Conceived, designed and writing / editing of manuscript.

***OW:*** Provision of Pathology images.

***JM:*** Provision of Radiology images.

***AH & FS:*** Review, editing and final approval of manuscript.
